# Biomarker-guided detection of acute kidney injury in abdominal aortic surgery: the new and the old

**DOI:** 10.3389/fmed.2024.1386018

**Published:** 2024-07-03

**Authors:** Christian Nusshag, Vivienne Theobald, Markus Wortmann, Philipp Kaimann, Maximilian Dietrich, Daniel Gruneberg, Kevin Tourelle, Maik von der Forst, Markus A. Weigand, Moritz S. Bischoff, Dittmar Böckler, Felix C. F. Schmitt

**Affiliations:** ^1^Department of Nephrology, Medical Faculty Heidelberg, Heidelberg University, Heidelberg, Germany; ^2^Department of Anesthesiology, Medical Faculty Heidelberg, Heidelberg University, Heidelberg, Germany; ^3^Department of Vascular, Endovascular Surgery and Transplant Surgery, Stuttgart Hospital, Stuttgart, Germany; ^4^Department of Vascular and Endovascular Surgery, University Hospital Heidelberg, Heidelberg, Germany

**Keywords:** acute kidney injury, aortic surgery, biomarkers, cell cycle arrest, intensive care medicine, soluble urokinase plasminogen activator receptor

## Abstract

**Introduction:**

Acute kidney injury (AKI) is a common complication in patients undergoing major vascular surgery. Despite significant research efforts in this area, the incidence of AKI remains high, posing a significant challenge to healthcare systems, especially in situations where resources are limited. Early prediction of AKI severity and individualized postoperative care is therefore essential.

**Methods:**

The primary objective of this exploratory study was to assess the diagnostic value of urine cell-cycle arrest biomarkers [(TIMP-2) × (IGFBP7)] and soluble urokinase plasminogen activator receptor (suPAR) for predicting moderate or severe AKI within 24 h after open aortic surgery, and compared to routine kidney biomarkers. Seventy-five patients undergoing elective aortic surgery were included. Clinical parameters, urine and blood samples were collected preoperatively, immediately postoperatively, and 24 h later. AKI was defined using KDIGO criteria. Individual and combined diagnostic performance of biomarkers were evaluated.

**Results:**

Of the 75 patients, 61% developed AKI, of which 28% developed moderate or severe AKI within 24 h of surgery. Baseline demographics, comorbidities and kidney parameters did not differ between patients with moderate or severe AKI (AKI II/III) and none or mild AKI (AKI 0/I), except for higher preoperative suPAR levels in later AKI II/III patients. Urine osmolality, Cystatin C and serum creatinine had the highest predictive power for AKI II/III with AUCs of 0.75–0.72. (TIMP-2) × (IGFBP7), and neither (TIMP-2) × (IGFBP7) nor suPAR individually showed superior diagnostic value. Combining CysC or SCr with urine osmolality and 6 h urine output gave the best performance with AUCs of 0.86 (95% CI, 0.74–0.96) and 0.85 (95% CI, 0.75–0.95) respectively.

**Conclusion:**

Our study suggests that routine parameters like urine osmolality, CysC, SCr and 6 h urine output perform best in predicting postoperative AKI after aortic surgery compared to the new biomarkers (TIMP-2) × (IGFBP7) and suPAR. Combining biomarkers, particularly CysC or SCr with urine output, urine osmolality, may enhance diagnostic accuracy. Further validation in larger cohorts and clinical settings is warranted to establish their clinical utility.

## Introduction

Acute kidney injury (AKI), a major postoperative complication, is particularly common in patients undergoing abdominal aortic surgery. The incidence of AKI in this cohort varies from 20 to 70%, a discrepancy that can be attributed to the heterogeneity of surgical procedures and the criteria used to define AKI (1). Patients from Western countries undergoing such surgical procedures are predominantly geriatric patients with a wide range of comorbidities, requiring close postoperative monitoring (1). In particular, the overlapping pathophysiological drivers of vascular disease and chronic progressive nephropathy, such as arterial hypertension, diabetes mellitus, and metabolic syndrome, explain why this patient population is at high risk for postoperative AKI (2).

The current gold standard for AKI grading are the 2012 Kidney Disease: Improving Global Outcomes classification (KDIGO) criteria, which use serum creatinine (SCr) and urine output as functional biomarkers to define AKI (3). Despite their generally proven clinical utility, the current KDIGO criteria have insufficient diagnostic accuracy for early detection of AKI and its later severity. This is partly due to a time latency between kidney injury and SCr maximum, with a typical SCr latency of 24 to 72 h between SCr peak and underlying kidney insult (4). Additional confounding factors affect blood SCr concentrations independent of kidney function or injury (4). These include individual variations in SCr production and tubular secretion, interference with the measurement method by endogenous metabolites or pharmacologic agents, and rapid changes in volume of distribution. In summary, these latter factors prevent early diagnosis and grading of AKI to adapt appropriate postoperative monitoring in patients after major vascular surgery (5, 6).

Newer, more direct kidney biomarkers of damage or stress, such as urinary insulin-like growth factor binding protein-7 (IGFBP-7) and tissue inhibitor of metalloproteinase-2 (TIMP-2) products, have shown promise in various contexts as tools for earlier detection of AKI. These biomarkers, which indicate tubular G1 cell cycle arrest, provide insight into the early phase of kidney tubular cell damage (7–9). A study by Kashani et al. (10) showed that the product of the two biomarkers IGFBP-7 and TIMP-2 [(TIMP-2) × (IGFBP7)] outperformed previously established biomarkers in predicting moderate to severe AKI within 12 h of ICU admission. This has led to the development of a point-of-care device, Nephrocheck^®^, to facilitate early detection of AKI (5, 11, 12).

In addition, soluble urokinase plasminogen activator receptor (suPAR), an immune system-derived biomarker, has been shown to be a potential predictor of AKI, particularly in acute but also in chronic systemic inflammatory conditions (13–16). Elevated blood suPAR levels are associated with a variety of organ dysfunctions, including kidney pathologies, and may be directly pathophysiologically related to the extent of organ-specific tissue inflammation, but also to vasculopathy, as recently published by Hindy et al. (16–19). Since chronic inflammatory conditions are a typical feature in patients with metabolic syndrome, diabetes mellitus, liver and cardiovascular disease, and these comorbidities are common in patients undergoing aortic surgery, suPAR could be an early risk stratifier for the appearance and severity of postoperative AKI in this context (15). However, the specific diagnostic accuracy of (TIMP-2) × (IGFBP-7) and suPAR (the new) for predicting AKI compared to established standard biomarkers (the old) is unknown in patients undergoing abdominal aortic surgery.

Therefore, this exploratory study aims to investigate the value of (TIMP-2) × (IGFBP7) and suPAR as diagnostic tools for early prediction of moderate or severe AKI within 24 h after open aortic surgery in comparison to routinely available kidney biomarkers. The goal of our study was to improve the accuracy of AKI prediction and detection to facilitate timely intervention in this high-risk surgical population and to personalize postoperative monitoring.

## Materials and methods

### Study design and patient population

The Roccet trial is was a prospective, exploratory, monocentric study to evaluate the role of urinary cell cycle arrest biomarkers and blood suPAR compared to widely available routine kidney standard parameters for early prediction of moderate or severe acute kidney injury within 24 h after aortic surgery. All patients underwent open abdominal aortic surgery and postoperative care at Heidelberg University Hospital, Germany. The study was approved by the Ethics Committee of the Medical Faculty of Heidelberg University Hospital did not object to the conduct of the study (internal number: S-270/2017). Written informed consent was obtained from all participants. The study complied with the principles of the Declaration of Helsinki in its current version.

Patients aged ≥18 years undergoing elective open abdominal aortic surgery were included. Exclusion criteria for all participants included lack of informed consent, absence of a permanent urinary catheter, pre-existing renal replacement therapy (RRT), or immediate RRT requirement on admission.

### Clinical investigations

Clinical parameters for patient characterization were obtained from medical records and standardized medical history forms at the time of enrollment and included demographics, comorbidities including cardiovascular, pulmonary, and renal disease, and reason for surgery. Urine laboratory data including urine creatinine, proteinuria, osmolality were collected at all time points of the study [preoperatively (pre), immediately postoperatively (d0), and the next morning (24 h postoperatively, d1)]. Fluid balance and urine output were measured during surgery and up to 24 h postoperatively. Data on type and duration of aortic clamping, intraoperative need for catecholamines, and number of hypotensive episodes during surgery were collected, as well as type of postoperative care and presence of organ failure using Acute Physiology And Chronic Health Evaluation (APACHE II) and, Simplified Acute Physiology Score II (SAPS II).

### Analysis of biomarkers

Kidney-specific biomarkers including SCr, Cystatin C (CysC), urine osmolality, urine α1-microglobulin urine output, urine creatinine, albuminuria, proteinuria, suPAR, TIMP2 and IGFBP7 were analyzed.

Serum and urine samples were obtained at pre, d0, and d1. Blood samples were centrifuged at 3,000 rounds per minute (rpm) for 10 and 15 min, respectively. The supernatants were immediately transferred to Eppendorf tubes and stored at −80°C. All samples were thawed directly prior to analysis. TIMP2 and IGFBP7 were measured using a commercially available, standardized point-of-care assay (NephroCheck^®^, Astute Medical, San Diego, CA, United States). Test results are given as product of both markers in (ng/mL)^2^/1,000. All other laboratory values including suPAR were measured in the accredited Central Laboratory of Heidelberg University Hospital.

### Definitions of endpoints and outcomes

The primary outcome measure was moderate or severe AKI (AKII/III) 24 h after surgery. AKI was determined 24 h postoperatively based on urine output and rise in SCr concentration from baseline according to the KDIGO criteria as follows (20):

- AKI I: increase of SCr by ≥0.3 mg/dL (≥26.4 μmol/L) or increase to ≥150–200% from baseline or urine output <0.5 mL/kg/h for >6 h.
- AKI II: increase of SCr to >200–300% from baseline and/or urine output <0.5 mL/kg/h for >12 h;
- AKI III: increase of SCr to >300% from baseline or SCr ≥4.0 mg/dL (≥354 μmol/L) after a rise of at least 44 μmol/L or treatment with renal replacement therapy and/or urine output <0.3 mL/kg/h for >24 h or anuria for 12 h.

Chronic kidney disease (CKD) was defined as estimated glomerular filtration rate (CKD-Epi eGFR) <60 mL/min/1.73 m^2^. Admission values of kidney parameters at the day prior elective surgery including SCr, CysC, (TIMP-2) × (IGFBP7), suPAR, proteinuria and α1-microglobulin were defined as baseline. Hypotensive episodes were defined as systolic blood pressure ≤20% of baseline for >5 min.

### Statistical methods

Statistical analyses were performed using SPSS Statistics 25 (IBM, Armonk, NY) and Graph Pad Prism 9 (GraphPad Software, La Jolla, CA). For all analyses, two-sided *p*-values of less than 0.05 were considered statistically significant. Receiver operating characteristics (ROCs) curves were generated to analyze individual biomarker performances. DeLong’s test was carried out for comparison of ROC curves. Logistic regression models were generated to assess an additive predictive value of biomarker combinations. This was performed as model I a/b: both functional biomarkers SCr (Ia) in combination with non-functional biomarkers and Cys C (Ib) in combination with non-functional biomarkers and in a second step as model IIa/b: both functional biomarkers Cys C (IIa) and SCr (IIb) together with urine output (KDIGO standard) and the non-functional biomarkers suPAR, (TIMP-2) × (IGFBP7), and urine osmolality. Continuous variables are presented as median (interquartile range); categoric variables are presented as numbers (%). Mann–Whitney *U* test was used for pairwise comparisons, and χ2 test for categorical variables.

## Results

### Patient characteristics and outcomes

**Figure 1:**
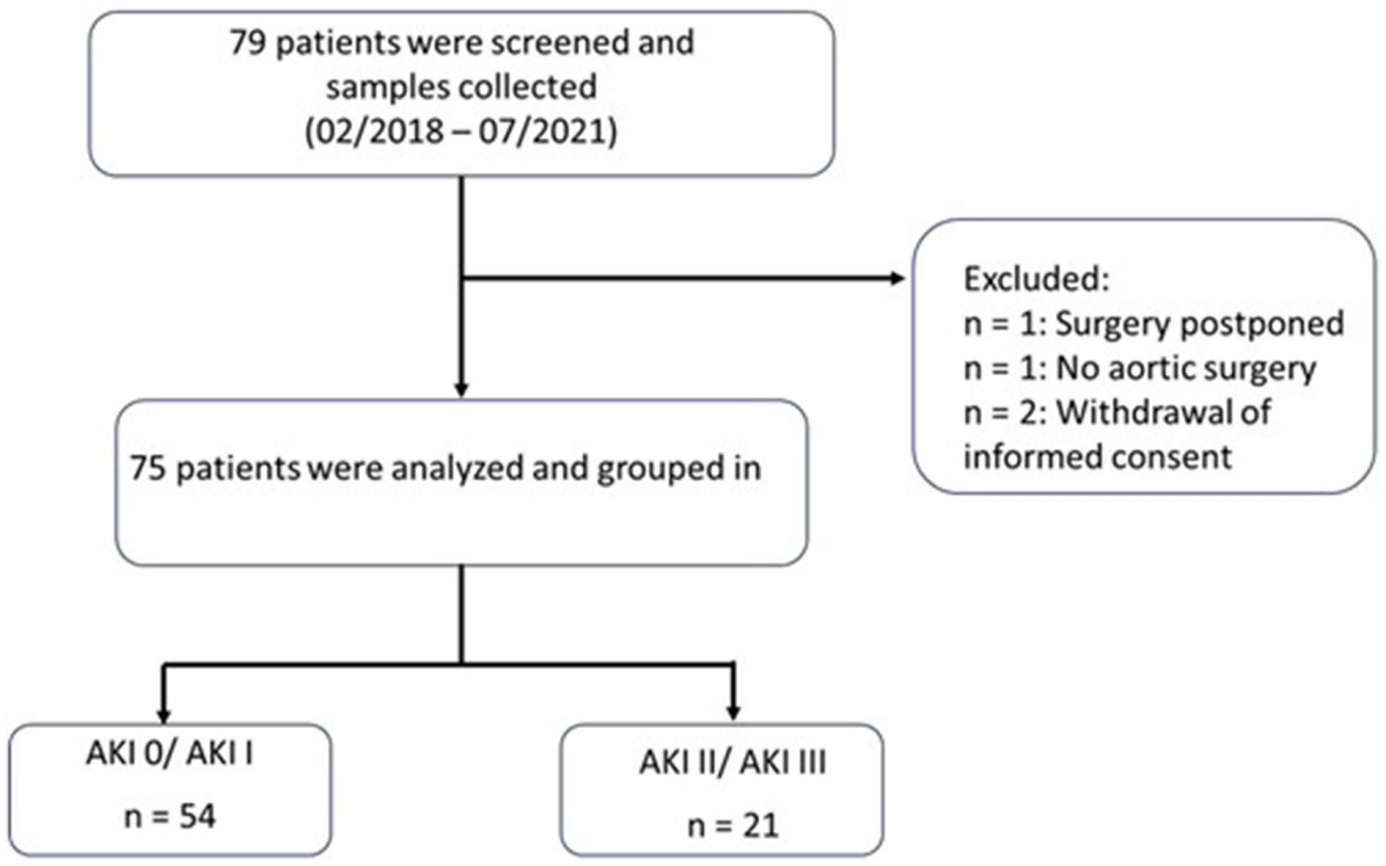
Flow chart of study design. AKI, acute kidney injury; KDIGO, Kidney Disease: Improving Global Outcomes classification.

For this study, 79 patients undergoing open abdominal aortic surgery between 05/02/2018 and 04/07/2021 were prospectively enrolled. One participant was excluded because surgery was postponed, another participant did not undergo aortic surgery, and two participants had to be excluded due to withdrawal of informed consent, leaving 75 patients for the final analysis (Figure 1). Of the 75 patients, 64 were male (85%). The median age was 67 (61–74) years. The overall AKI incidence in our cohort was 61%.

Table 1 shows the baseline characteristics of the entire study cohort comparing patients without AKI or AKI I and patients with AKI II and III. Twenty-one patients met the primary outcome of AKI II/III 24 h after surgery (Figure 1). With the exception of preoperative suPAR levels, baseline demographics, preexisting comorbidities and baseline preoperative kidney parameters did not differ between the two groups. Surgery time was significantly longer in patients with later AKI II/III. However, cross-clamp type (supra-/infrarenal), cross-clamp time, intraoperative blood loss, urine output, cumulative vasopressor dose and number of hypotensive episodes were comparable between patients with later AKI 0/I and AKI II/III. The transfusion rate was higher in patients with AKI II/III. Overall estimated blood loss was 1 L. Intraoperarive fluid balance was higher in patients with AKI II/III. The need for vasopressors and RRT after surgery was more common in patients with AKI II/III. Accordingly, urine output was lower and length of ICU stay was longer in patients who evolved AKI II/III. In contrast, disease severity scores and mortality did not differ between groups. The 30-day all-cause mortality was 1.33% (no deaths in the first 7 days). Only one patient in the AKI II/III group died within 30 days of enrollment, but did not require RRT (Table 1).

**Table 1:** Baseline characteristics and outcomes.

Variable	All patients (*n* = 75)	No AKI/AKI I (*n* = 54)	AKI II/AKI III (*n* = 21)	*p*-value
**Demographics**				
Age, years	67.00 (61.00–74.00)	69.00 (63.00–75.00)	65.00 (60.50–70.50)	0.098
Male gender, *n* (%)	64 (85.3)	46 (85.2)	18 (85.7)	0.954
BMI, kg/m^2^	26.42 (23.29–28.72)	25.93 (23.55–29.41)	26.70 (22.11–28.37)	0.571
**Pre-existing comorbidities, *n* (%)**				
Chronic kidney disease (CKD-Epi eGFR <60 mL/min/1.73 m^2^)	8 (10.7)	6 (11.1)	2 (9.5)	0.269
Hypertension	60 (80.0)	44 (81.5)	16 (76.2)	0.607
Diabetes mellitus	20 (26.7)	13 (24.1)	7 (33.3)	0.416
Coronary heart disease	39 (52)	31 (57.4)	8 (38.1)	0.133
Peripheral arterial disease	29 (38.7)	22 (40.7)	7 (33.3)	0.554
Congestive heart failure	19 (25.3)	14 (25.9)	5 (23.8)	0.850
Chronic obstructive pulmonary disease	17 (22.7)	11 (20.4)	6 (28.6)	0.446
**Preoperative kidney parameters**				
SCr, mg/dL	0.82 (0.73–1.00)	0.83 (0.73–0.98)	0.8 (0.71–1.00)	0.777
Cystatin C, mg/L	1.07 (0.95–1.33)	1.08 (0.95–1.34)	1.06 (0.96–1.33)	0.813
(TIMP-2) × (IGFBP7), (ng/mL)^2^/1000	0.15 (0.08–0.44)	0.15 (0.08–0.44)	0.21 (0.07–0.48)	0.572
suPAR, ng/mL	3.23 (2.67–3.91)	3.11 (2.62–3.82)	3.62 (3.06–4.12)	**0.046**
Proteinuria, g/L	0.05 (0.02–0.19)	0.05 (0.02–0.14)	0.07 (0.04–0.25)	0.119
α1-microglobulin, mg/L	6.30 (0.00–14.33)	6.20 (0.00–14.8)	7.3 (0.00–9.00)	0.310
**Surgery and intraoperative parameters**				
*Reason for surgery, n (%)*				
Aortic aneurysm	62 (82.7)	45 (83.3)	17 (81.0)	0.768
Aorto-iliac occlusive disease	8 (10.7)	5 (9.3)	3 (14.3)	
Graft infection	5 (6.7)	4 (7.4)	1 (4.8)	
Elective surgery, *n* (%)	75 (100)	54 (100)	21 (100)	1.00
Duration surgery, min	253.00 (178.00–284.00)	210.00 (178.75–270.50)	280.00 (178.00–400.00)	**0.027**
*Type of clamping*				
None	1 (1.3)	1 (1.9)	0 (0)	0.704
Suprarenal	35 (46.7)	24 (44.4)	11 (52.4)	
Infrarenal	39 (52.0)	29 (53.7)	10 (47.6)	
Cross clamp time, min	35.00 (27.00–56.00)	33.00 (25.00–51.25)	50.00 (31.00–60.00)	0.056
Blood loss, L	1.00 (0.6–1.6)	0.96 (0.6–1.5)	1.10 (0.7–2.5)	0.066
Blood transfusion, *n* (%)	17 (22.7)	8 (14.8)	9 (42.9)	**0.036**
Urine output, L	0.50 (0.20–0.80)	0.50 (0.28–0.86)	0.51 (0.05–0.80)	0.670
NA intraoperative, μg	1140.00 (600.00–1900.00)	1066.75 (575.00–1755.00)	1468.00 (843.10–2490.00)	0.139
Hypotensive episodes, *n* (%) (systolic BP ≤20% of baseline for >5 min)	32 (42.7)	19 (35.2)	13 (61.9)	0.246
Fluid intake, L	4.91 (1.5–10.7)	4.55 (1.50–8.90)	5.82 (2.00–10.7)	**0.018**
Fluid balance, L	3.09 (0.60–7.40)	2.82 (0.60–6.06)	3.78 (1.03–7.40)	**0.016**
**Postoperative scores**				
SAPS II	16.00 (12.00–18.00)	16.00 (12.00–18.00)	16.00 (12.00–19.00)	0.717
APACHE II	7.00 (5.00–9.00)	6.50 (5.00–8.25)	8.00 (5.00–9.00)	0.390
**Outcomes**				
Urine output 6 h postoperative, L	0.61 (0.00–3.92)	0.70 (0.00–3.92)	0.39 (0.00–1.30)	**0.013**
Postoperative vasopressor support, *n* (%)	36 (48)	21 (38.9)	15 (71.4)	**0.011**
Need for RRT, *N* (%)	2 (2.67)	0 (0.00)	2 (9.52)	**0.022**
Length of ICU stay, days	1.00 (1.00–4.00)	1.00 (1.00–2.00)	3.00 (1.50–6.00)	**0.005**
Length of hospital stay, days	13.00 (11.00–17.00)	13.00 (11.00–16-25)	13.00 (11.00–17.00)	0.627
Mortality 7 days, *n* (%)	0.00 (0.00)	0.00 (0.00)	0.00 (0.00)	1.00
Mortality 30 days, *n* (%)	1.00 (1.33)	0 (0.00)	1 (4.76)	0.106

APACHE II, Acute Physiology and Chronic Health Evaluation II; BMI, body mass index; BP, blood pressure; ICU, intensive care unit; NA, noradrenaline; RRT, renal replacement therapy; SAPS II, Simplified Acute Physiology Score II; SCr, serum creatinine; suPAR, soluble urokinase plasminogen activator receptor; SOFA, Sequential Organ Failure Assessment.Data are reported as median (IQR) unless otherwise indicated and as n and %.Bold values are statistically significant for *p* ≤ 0.05.

### Longitudinal kinetics of kidney biomarkers stratified by AKI severity

**Figure 2:**
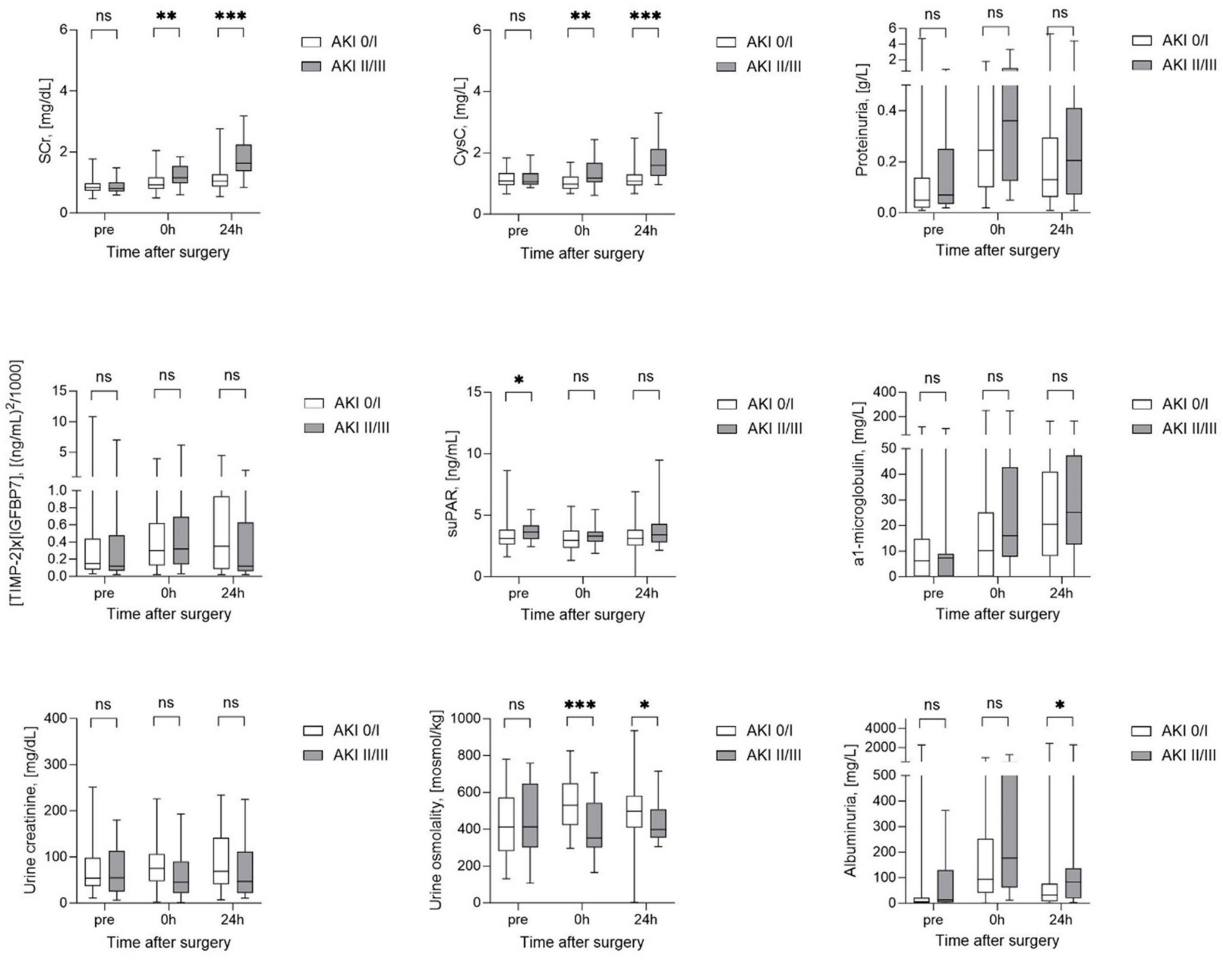
Longitudinal biomarker characteristics stratified by AKI severity. AKI, acute kidney injury; CysC, Cystatin C; SCr, serum creatinine; suPAR, soluble urokinase plasminogen activator receptor. ^*^Statistically significant for *p* ≤ 0.05; ^**^statistically significant for *p* ≤ 0.01; ^***^statistically significant for *p* ≤ 0.001.

SCr, CysC, proteinuria, albuminuria as well as urine osmolality, creatinine and α1-microglobulin, levels did not differ between groups before surgery (Figure 2). The same was true for preoperative urinary levels of the new kidney stress biomarker (TIMP-2) × (IGFBP7), while preoperative blood suPAR levels were already significantly higher in patients with later AKI II/III compared to patients with AKI 0/I. Immediately postoperative as well as 24 h later (24 h), levels of SCr, CysC were higher and especially urine osmolality was significantly lower in patients with AKI II/III compared to AKI 0/I. In contrast, proteinuria, albuminuria, urine creatinine and α1-microglobulin as well as (TIMP-2) × (IGFBP7) and suPAR did not allow an postoperative differentiation between the two groups AKI 0/I and AKI II/III (Figure 2). Only, albuminuria was significantly higher at 24 h after surgery in patients with AKI II/III, when the primary endpoint of the study was already achieved.

### Diagnostic performance of kidney biomarkers and their complementary role for outcome prediction

Table 2 shows the performance of all tested biomarkers immediately postoperative with Area under the receiver operator curve (AUC) above 0.50 ranked from best to worst performance. In descending order, urine osmolality, Cys C and SCr showed the highest AUCs in predicting AKI II/III within 24 h with AUCs ranging from 0.75–0.72. In addition, postoperative urine output within the first 6 h after surgery showed a lower AUC of 0.69 (95% CI, 0.55–0.82).

**Table 2:** Diagnostic value of individual biomarkers measured immediately after surgery to predict moderate or severe AKI within 24 h.

Biomarker	AUC	95% CI	*p*-value
Urine osmolality, (mosmol/kg)	0.75	0.62–0.88	**0.001**
CysC, (mg/L)	0.73	0.60–0.87	**0.002**
SCr, (mg/dL)	0.72	0.59–0.85	**0.003**
Postoperative urine output first 6 h, (L)	0.69	0.55–0.82	**0.013**
Urine creatinine, (mg/dL)	0.64	0.49–0.79	0.059
Albuminuria, (mg/L)	0.64	0.50–0.79	0.054
α1-microglobulin, (mg/L)	0.63	0.48–0.78	0.089
Proteinuria, (g/L)	0.62	0.47–0.77	0.099
suPAR, (ng/mL)	0.59	0.45–0.73	0.233
(TIMP-2) × (IGFBP7), [(ng/mL)^2^/1000]	0.53	0.38–0.68	0.693

AUC, area under the receiver operator curve; CysC, Cystatin C; SCr, serum creatinine; suPAR, soluble urokinase plasminogen activator receptor.Bold values are statistically significant for *p* ≤ 0.05.

In contrast, suPAR and (TIMP-2) × (IGFBP7) as well as urine creatinine, albuminuria, α1-microglobulin did not allow meaningfull and early identification of patients at risk for AKI II/III (Table 2).

To further investigate a potential complementary role of biomarkers for outcome prediction, the two kidney function biomarkers CysC and SCr were individually combined with the non-function biomarkers postoperative urine output first 6 h, urine osmolality, urine creatinine, albuminuria, proteinuria, α1-microglobulin, suPAR and (TIMP-2) × (IGFBP7) (Table 3). The highest diagnostic accuracy was achieved by combining CysC and SCr individually with the widely available parameter urine osmolality and postoperative urine output first 6 h (Table 3). The latter AUCs were higher than when biomarkers were tested alone. In general, higher AUCs were observed for CysC in combination with other tested biomarkers compared to combinations with SCr. The second highest AUCs, again superior to biomarkers alone, were found for CysC and SCr in combination with albuminuria, followed by the combination of CysC with proteinuria and urine creatinine. The combination of SCr with suPAR and proteinuria were on par with CysC together with α1-microglobulin and suPAR. The combination of CysC and SCr with [TIMP-2]x[IGFBP7] had no additional diagnostic value compared to CysC and SCr alone (Table 3).

**Table 3:** Diagnostic value of biomarker combinations measured immediately after surgery to predict moderate or severe AKI within 24 h.

Biomarker		AUC	95% CI	*p*-value
Model Ia: SCr +	Urine osmolality, (mosmol/kg)	0.79	0.67–0.91	**<0.001**
	Postoperative urine output first 6 h, (L)	0.79	0.69–0.89	**<0.001**
	Albuminuria, (mg/L)	0.75	0.62–0.88	**0.001**
	suPAR, (ng/mL)	0.74	0.62–0.86	**0.001**
	Proteinuria, (g/L)	0.74	0.61–0.87	**0.001**
	α1-microglobulin, (mg/L)	0.73	0.60–0.86	**0.002**
	(TIMP-2) × (IGFBP7), [(ng/mL)^2^/1000]	0.71	0.58–0.85	**0.004**
Model Ib: CysC +	Postoperative urine output first 6 h, (L)	0.82	0.72–0.92	**<0.001**
	Urine osmolality, (mosmol/kg)	0.81	0.69–0.93	**<0.001**
	Albuminuria, (mg/L)	0.78	0.65–0.91	**<0.001**
	Proteinuria, (g/L)	0.76	0.63–0.90	**<0.001**
	Urine creatinine, (mg/dL)	0.76	0.62–0.89	**0.001**
	α1-microglobulin, (mg/L)	0.74	0.60–0.87	**0.001**
	suPAR, (ng/mL)	0.74	0.60–0.87	**0.002**
	(TIMP-2) × (IGFBP7), [(ng/mL)^2^/1000]	0.73	0.59–0.87	**0.003**

AUC, area under the receiver operator curve; CysC, Cystatin C; SCr, serum creatinine; suPAR, soluble urokinase plasminogen activator receptor.Bold values are statistically significant for *p* ≤ 0.05.

**Figure 3:**
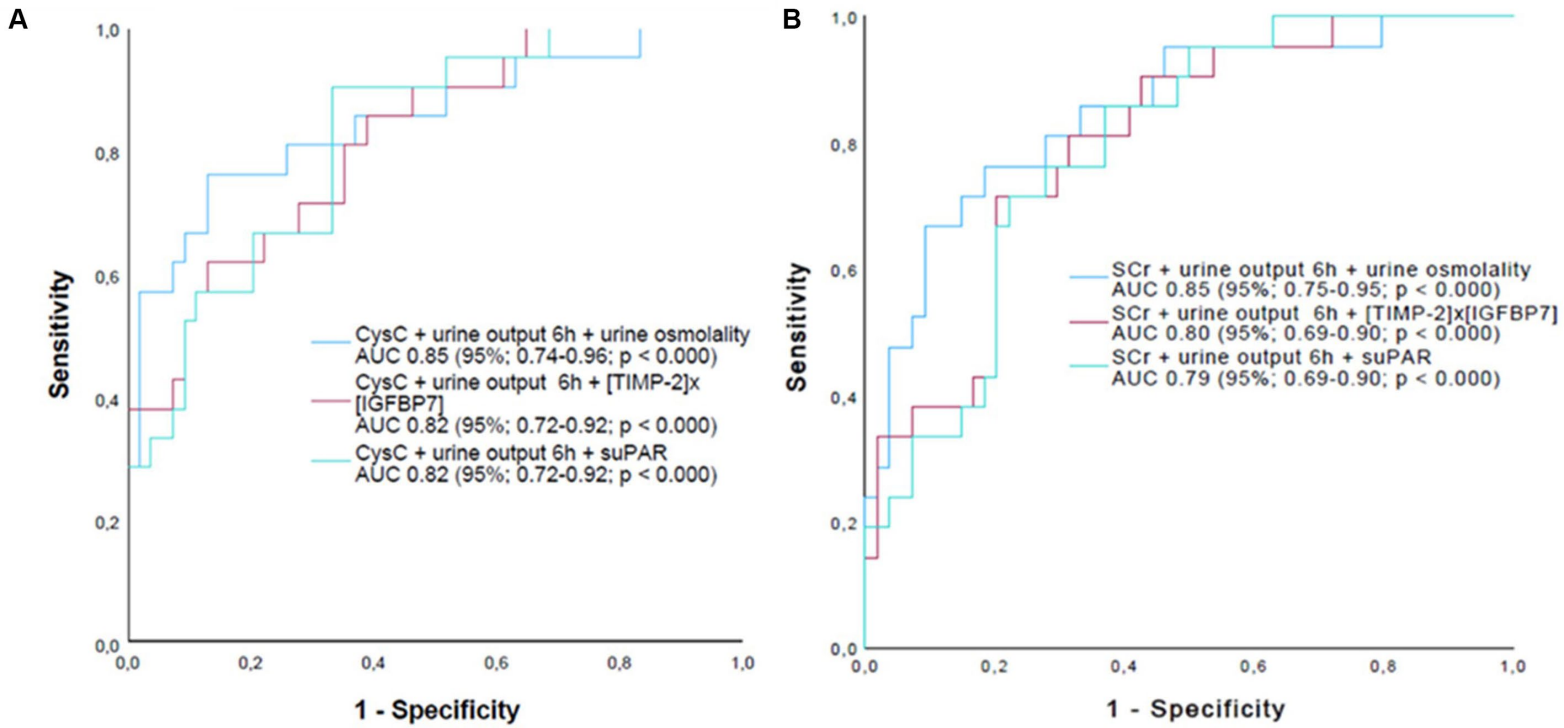
AUC curves of biomarker triplet combinations measured immediately after surgery to predict moderate or severe AKI within 24 h. **(A)** Biomarker triplet combinations with CysC. **(B)** Biomarker triplet combinations with SCr. AKI, acute kidney injury; CysC, Cystatin C; SCr, serum creatinine; suPAR, soluble urokinase plasminogen activator receptor. Urine ouput 6 h postoperatively.

Finally, to investigate a potential additive diagnostic value of the newly tested biomarkers to the currently established biomarkers of AKI staging (function biomarker + urine output) a second model for predicting AKI II/III combining SCr and urine output 6 h or CysC and urine output 6 h with urine osmolality, (TIMP-2) × (IGFBP7) or suPAR was performed (Figure 3).

While including urine osmolality in the model further improved absolute AUCs compared to combining SCr or CysC with urine output alone, there was no additional improvement when (TIMP-2) × (IGFBP7) or suPAR were included in the model instead of urine osmolality. However, despite the fact that absolute AUC values improved when urine osmolality was added to the model, the AUC of 0.85 achieved by combining SCr, 6 h urine output and urine osmolality failed to accomplish statistical superiority (de Long’s test) compared with the dual combinations of SCr with urine output 6 h (*p* = 0.109) or urine osmolality (*p* = 0.060). On the other hand, the combination of SCr, 6 h urine output and urine osmolality was statistically superior to the dual combination of SCr with (TIMP-2) × (IGFBP7) (*p* = 0.044) or suPAR (*p* = 0.048). In contrast, the dual combination of SCr and urine output 6 h alone did not reach statistical superiority over the dual combination of SCr with (TIMP-2) × (IGFBP7) (*p* = 0.219) or suPAR (*p* = 0.339).

## Discussion

Patients after open abdominal aortic surgery are at high risk for postoperative AKI (21) and this goes along with increased morbidity and mortality, prolonged ICU stays and reduced kidney recovery upon hospital discharge (22). Despite these consequences, to characterize the extent and progression of kidney damage accurately and early using current standards remains a challenge in this population. However, the latter is of great relevance in order to take adequate therapeutic measures at an early stage and to adapt postoperative monitoring. On the other hand, early triage between patients who develop morderate or severe AKI and patients with no or mild AKI risk can save healthcare resources, as this enables early transfer to a normal ward. Accordingly, the development and validation of novel kidney damage/stress biomarkers such as (TIMP-2) × (IGFBP7) has been driven forward in recent years (22). However there is limited data on the comparison with widely available kidney biomarkers beyond SCr and urinary output, such as albuminuria, proteinuria, urine osmolality and others. This makes a conclusive assessment of the clinical relevance of these newly developed damage biomarkers difficult, even though their mostly direct involvement in AKI pathophysiology in general represents a promising conceptual extension to the purely functional indicators SCr, CysC and urinary output.

Our current study now surprisingly shows that an immediate postoperative determination (0 h) of the already widely accessible kidney biomarkers urine osmolality, 6 h postoperative urine output and SCr or plasma CysC, in comparison to the novel markers suPAR and (TIMP-2) × (IGFBP7), provide the best value regarding the prediction of AKI II/III within 24 h after aortic surgery. Considering all biomarkers individually, urine osmolality showed the highest AUC predicting subsequent AKI II/III. As shown in other studies, this may suggest the relevance of tubular apparatus dysfunction and a resulting tubular concentration defect for AKI after aortic surgery (23). However, the prognostic and pathophysiological significance of urine osmolality for AKI and tubular damage in general is not yet fully understood (23, 24). Other factors independent of tubule damage may contribute to changes in urine osmolality, such as changes in antidiuretic hormone (ADH) secretion caused by intraoperative volume shifts, blood pressure fluctuations, the consumption of noradrenaline and the type of volume replacement solution used (25). This may also be the case in our study since α1-microglobulin, another tubular damage biomarker (26), showed no longitudinal differences depending on the severity of AKI. In addition, data by Amatrura et al. (27) showed that postoperative α1-microglobulin did not appear to be informative for the prediction of AKI in patients after cardiac surgery. Nevertheless, in our study, vasopressor use did not differ between the two groups and patients were treated with the same crystalloid fluid. It is however worth noting that patients with AKI II/III had significantly higher intraoperative fluid intake and fluid balance than patients with AKI 0/I, possibly due to longer duration of surgery. Therefore, higher intraoperative fluid administration may be partly responsible for the lower urine osmolality in patients with later AKI II/III. However, urine output 6 h postoperatively shows that the urine volume in patients with late AKI II/III was rather reduced compared to AKI 0/I—as expected for more severe AKI—which contradicts a simple dilution of urine due to increased fluid intake. Thus, as the addition of urine osmolality to our models, which already included urine output, improved outcome prediction performance further, urine osmolality appears to have additional diagnostic information beyond a mere dilutive component based on higher fluid administration. It remains to be seen what tubular damage can be detected with urine osmolality, which does not appear to have any influence on the change in α1-microglobulin. Preclinical studies could help to clarify the causes and support the later use of urine osmolality in clinical practice. However, it must be emphasized here that there are clear differences in the development of biomarker-based diagnostics in preclinical studies and the implementation of clinical studies based on them in the literature. Biomarkers are more frequently used as primary endpoints in preclinical research than in clinical studies. Such differences make the implementation of biomarkers more difficult, as their benefits are often not tested to the same extent in the clinical practice (28).

CysC is an alternative kidney function biomarker to SCr with potentially superior diagnostic properties such as faster kinetics and independence from muscle mass. Consistent with data from different clinical contexts, including AKI after cardiac surgery, CysC also showed promising performance in the postoperative prediction of AKI II/III in our study (29–31).

Nevertheless, the AUC of SCr for predicting AKI II/III was only slightly lower compared to CysC indicating that SCr kinetics should not be undererstimated in the postoperative context. A similar observation for SCr was recently made by Pilarczyk et al. (31) in patients after vascular surgery, showing an excellent AUC of 0.82 (95% CI; 0.73–0.92) and 0.89 (95% CI; 0.78–1.0) after 2 and 6 h, respectively, to predict moderate or severe AKI 48 h after surgery.

Interestingly, the mean levels of urinary indicators for total, glomerular, and tubular proteinuria (proteinuria, albuminuria, and alpha1-microglobulin) increased regardless of the later stage of AKI and compared to preoperative levels and may reflect impaired tubular or glomerular integrity, or glomerular filtration pressure variation. However, the short- and long-term relevance and consequences of different types of proteinuria in AKI has not been sufficiently investigated. In our study, they were not suitable for early prediction of AKI II/III, when considering only the individual biomarkers.

The same holds true for urinary creatinine concentrations. Though a tendency towards lower levels was observed postoperatively in patients with later AKI II/III, this difference showed a poor diagnostic value. In this setting, however, a lower urine output in patients with AKI II/III may have influenced urinary creatinine towards higher concentrations (32, 33). Further, neither postoperative (TIMP-2) × (IGFBP7) nor suPAR levels showed any additional diagnostic value compared to widely available standard kidney biomarkers. However, with regard to (TIMP-2) × (IGFBP7), the literature is highly inconclusive. While the results of Finge et al. (34) and Pilarczyk et al. (31) are consistent with our data and show poor diagnostic value for AKI prediction after cardiac and thoracic aortic surgery, studies in more heterogeneous cohorts after major surgery have shown very promising results (35, 36). This potentially highlights a context-dependent value of kidney stress and damage biomarkers in daily routine.

As the only biomarker prior to surgery, suPAR was already significantly higher in patients with later AKI II/III compared to patients with AKI 0/I. This strengthens the concept of suPAR as an AKI risk stratifier prior to a potentially kidney harming event, as recently shown in a large study with different AKI entities and in a study in patients after cardiac surgery (14, 37). In these patients, suPAR could serve as an indicator of pre-existing acute or chronic inflammatory conditions as a risk factor for the development of AKI (13, 14, 19). In the present study, however, no correlation was found between postoperative suPAR levels and the extent of AKI. This may indicate that surgically induced inflammation is not a major factor in the pathophysiology of postoperative AKI, in contrast to the inflammatory states caused by sepsis (19, 38). Lastly, the combination of the functional biomarkers SCr and CysC with especially urine osmolality, 6 h urine excretion, or surprisingly albuminuria and proteinuria showed additional diagnostic value compared to the biomarkers alone. Furthermore, in contrast to (TIMP-2) × (IGFBP-7) and suPAR, the addition of urine osmolality to a model including CysC or SCr together with urine output 6 h had an additive diagnostic value with an improvement in absolute AUCs to predict AKI II/III. However, the triple combination of SCr, urine output and urine osmolality failed to show statistical superiority over a dual combination of SCr with urine output or urine osmolality, possibly due to the small sample size of our study. On the other hand, the triple combination was indeed statistically superior to the combination of SCr with (TIMP-2) × (IGFBP-7) or suPAR, whereas the dual combination of SCr and urine output 6 h alone failed to show statistical over the dual combination of SCr with (TIMP-2) × (IGFBP-7) or suPAR, highlighting the once more the additional diagnostic value of urine osmolality.

Thus, our observations underline the potential importance of combining currently used biomarkers of function and AKI staging with other directly pathophysiologically involved biomarkers to improve clinical decision making such as postoperative monitoring after aortic surgery in the future.

In conclusion, urine osmolality appears to be a relevant prognostic indicator with a potential additive diagnositc value for the detection of AKI II/III in patients undergoing open aortic surgery. To improve the predictive accuracy of postoperative AKI, a combination of CysC or SCr with urine output and urine osmolality should be considered in future investigations and study designs. In contrast, recently introduced biomarkers such as (TIMP-2) × (IGFBP-7) and suPAR showed limited postoperative value for predicting AKI II/III in our study cohort. Especially for suPAR, this may indicate a less important role of inflammatory drivers in the postoperative phase of aortic surgery induced AKI. Based on the inconclusive literature regarding (TIMP-2) × (IGFBP-7), further investigation is essential to clarify its context-specific significance. In addition, the role of serial measurements must be discussed at this point. A study by Fiorentino et al. (39) showed that in patients with septic shock, the values of (TIMP-2) × (IGFBP-7) differed according to the resuscitation phase. Patients with elevated biomaker values despite haemodynamic stabilization had a poorer outcome. Accordingly, future biomaker studies should examine the dynamics of biomakers over several days and take into account the influence of factors that improve renal function like the administration of fluids and hemodynamic stabilization. The exploratory nature of our study is a limitation that needs to be addressed. Therefore, the performance of the tested biomarkers needs to be validated in larger cohorts and in clinical routine.

## Data availability statement

The raw data supporting the conclusions of this article will be made available by the authors, without undue reservation.

## Ethics statement

The studies involving humans were approved by Ethics Committee of the Medical Faculty of Heidelberg University Hospital. The studies were conducted in accordance with the local legislation and institutional requirements. The participants provided their written informed consent to participate in this study.

## Author contributions

CN: Conceptualization, Data curation, Formal analysis, Funding acquisition, Investigation, Methodology, Project administration, Resources, Software, Supervision, Validation, Visualization, Writing – original draft, Writing – review & editing. VT: Conceptualization, Data curation, Formal analysis, Funding acquisition, Investigation, Methodology, Project administration, Resources, Software, Supervision, Visualization, Writing – original draft, Writing – review & editing. MWo: Investigation, Writing – review & editing. PK: Investigation, Methodology, Writing – review & editing. MD: Investigation, Writing – review & editing. DG: Investigation, Writing – review & editing. KT: Investigation, Writing – review & editing. MF: Investigation, Writing – review & editing. MWe: Investigation, Supervision, Writing – review & editing. MB: Investigation, Writing – review & editing. DB: Investigation, Writing – review & editing. FS: Conceptualization, Funding acquisition, Investigation, Methodology, Project administration, Resources, Writing – review & editing.

## References

[1] Nadim MK Forni LG Bihorac A Hobson C Koyner JL Shaw A. Cardiac and vascular surgery-associated acute kidney injury: the 20th international consensus conference of the ADQI (acute disease quality initiative) group. J Am Heart Assoc. (2018) 7:e008834. doi: 10.1161/JAHA.118.008834, PMID: 29858368 PMC6015369

[2] Hobson C Lysak N Huber M Scali S Bihorac A. Epidemiology, outcomes, and management of acute kidney injury in the vascular surgery patient. J Vasc Surg. (2018) 68:916–28. doi: 10.1016/j.jvs.2018.05.017, PMID: 30146038 PMC6236681

[3] Kellum JA Lameire N. Diagnosis, evaluation, and management of acute kidney injury: a KDIGO summary (part 1). Crit Care. (2013) 17:204. doi: 10.1186/cc11454, PMID: 23394211 PMC4057151

[4] Thomas ME Blaine C Dawnay A Devonald MA Ftouh S Laing C. The definition of acute kidney injury and its use in practice. Kidney Int. (2015) 87:62–73. doi: 10.1038/ki.2014.328, PMID: 25317932

[5] Endre ZH Pickering JW. Acute kidney injury: cell cycle arrest biomarkers win race for AKI diagnosis. Nat Rev Nephrol. (2014) 10:683–5. doi: 10.1038/nrneph.2014.198, PMID: 25347946

[6] Moran SM Myers BD. Course of acute renal failure studied by a model of creatinine kinetics. Kidney Int. (1985) 27:928–37. doi: 10.1038/ki.1985.101 4021321

[7] Boonstra J Post JA. Molecular events associated with reactive oxygen species and cell cycle progression in mammalian cells. Gene. (2004) 337:1–13. doi: 10.1016/j.gene.2004.04.032, PMID: 15276197

[8] Rodier F Campisi J Bhaumik D. Two faces of p53: aging and tumor suppression. Nucleic Acids Res. (2007) 35:7475–84. doi: 10.1093/nar/gkm744, PMID: 17942417 PMC2190721

[9] Seo DW Li H Qu CK Oh J Kim YS Diaz T. Shp-1 mediates the antiproliferative activity of tissue inhibitor of metalloproteinase-2 in human microvascular endothelial cells. J Biol Chem. (2006) 281:3711–21. doi: 10.1074/jbc.M509932200, PMID: 16326706 PMC1361361

[10] Kashani K Steuernagle JH Akhoundi A Alsara A Hanson AC Kor DJ. Vascular surgery kidney injury predictive score: a historical cohort study. J Cardiothorac Vasc Anesth. (2015) 29:1588–95. doi: 10.1053/j.jvca.2015.04.013, PMID: 26159745

[11] Bihorac A Chawla LS Shaw AD Al-Khafaji A Davison DL Demuth GE. Validation of cell-cycle arrest biomarkers for acute kidney injury using clinical adjudication. Am J Respir Crit Care Med. (2014) 189:932–9. doi: 10.1164/rccm.201401-0077OC, PMID: 24559465

[12] Hoste EA McCullough PA Kashani K Chawla LS Joannidis M Shaw AD. Derivation and validation of cutoffs for clinical use of cell cycle arrest biomarkers. Nephrol Dial Transplant. (2014) 29:2054–61. doi: 10.1093/ndt/gfu292, PMID: 25237065 PMC4209880

[13] Nusshag C Rupp C Schmitt F Krautkrämer E Speer C Kälble F. Cell cycle biomarkers and soluble urokinase-type plasminogen activator receptor for the prediction of sepsis-induced acute kidney injury requiring renal replacement therapy: a prospective, exploratory study. Crit Care Med. (2019) 47:e999–e1007. doi: 10.1097/CCM.0000000000004042, PMID: 31584458 PMC6867703

[14] Hayek SS Leaf DE Samman Tahhan A Raad M Sharma S Waikar SS. Soluble urokinase receptor and acute kidney injury. N Engl J Med. (2020) 382:416–26. doi: 10.1056/NEJMoa1911481 31995687 PMC7065830

[15] Rasmussen LJH Petersen JEV Eugen-Olsen J. Soluble urokinase plasminogen activator receptor (suPAR) as a biomarker of systemic chronic inflammation. Front Immunol. (2021) 12:780641. doi: 10.3389/fimmu.2021.780641, PMID: 34925360 PMC8674945

[16] Hindy G Tyrrell DJ Vasbinder A Wei C Presswalla F Wang H. Increased soluble urokinase plasminogen activator levels modulate monocyte function to promote atherosclerosis. J Clin Invest. (2022) 132:e158788. doi: 10.1172/JCI158788, PMID: 36194491 PMC9754000

[17] Koch A Zimmermann HW Gassler N Jochum C Weiskirchen R Bruensing J. Clinical relevance and cellular source of elevated soluble urokinase plasminogen activator receptor (suPAR) in acute liver failure. Liver Int. (2014) 34:1330–9. doi: 10.1111/liv.12512, PMID: 24575897

[18] Mondino A Blasi F. uPA and uPAR in fibrinolysis, immunity and pathology. Trends Immunol. (2004) 25:450–5. doi: 10.1016/j.it.2004.06.004, PMID: 15275645

[19] Nusshag C Wei C Hahm E Hayek SS Li J Samelko B. suPAR links a dysregulated immune response to tissue inflammation and sepsis-induced acute kidney injury. JCI Insight. (2023) 8:e165740. doi: 10.1172/jci.insight.165740, PMID: 37036003 PMC10132159

[20] Khwaja A. KDIGO clinical practice guidelines for acute kidney injury. Nephron Clin Pract. (2012) 120:c179–84. doi: 10.1159/000339789 22890468

[21] Zabrocki L Marquardt F Albrecht K Herget-Rosenthal S. Acute kidney injury after abdominal aortic aneurysm repair: current epidemiology and potential prevention. Int Urol Nephrol. (2018) 50:331–7. doi: 10.1007/s11255-017-1767-8, PMID: 29230707

[22] Nalesso F Cattarin L Gobbi L Fragasso A Garzotto F Calò LA. Evaluating Nephrocheck^®^ as a predictive tool for acute kidney injury. Int J Nephrol Renovasc Dis. (2020) 13:85–96. doi: 10.2147/IJNRD.S198222, PMID: 32425580 PMC7189184

[23] Hebert LA Greene T Levey A Falkenhain ME Klahr S. High urine volume and low urine osmolality are risk factors for faster progression of renal disease. Am J Kidney Dis. (2003) 41:962–71. doi: 10.1016/S0272-6386(03)00193-8, PMID: 12722030

[24] Lee MJ Chang TI Lee J Kim YH Oh KH Lee SW. Urine osmolality and renal outcome in patients with chronic kidney disease: results from the KNOW-CKD. Kidney Blood Press Res. (2019) 44:1089–100. doi: 10.1159/000502291, PMID: 31505490

[25] Klein JD Murrell BP Tucker S Kim YH Sands JM. Urea transporter UT-A1 and aquaporin-2 proteins decrease in response to angiotensin II or norepinephrine-induced acute hypertension. Am J Physiol Renal Physiol. (2006) 291:F952–9. doi: 10.1152/ajprenal.00173.2006, PMID: 16788141

[26] Penders J Delanghe JR. Alpha 1-microglobulin: clinical laboratory aspects and applications. Clin Chim Acta. (2004) 346:107–18. doi: 10.1016/j.cccn.2004.03.037 15256311

[27] Amatruda JG Estrella MM Garg AX Thiessen-Philbrook H McArthur E Coca SG. Urine alpha-1-microglobulin levels and acute kidney injury, mortality, and cardiovascular events following cardiac surgery. Am J Nephrol. (2021) 52:673–83. doi: 10.1159/000518240, PMID: 34515046 PMC8619798

[28] Fiorentino M Castellano G Kellum JA. Differences in acute kidney injury ascertainment for clinical and preclinical studies. Nephrol Dial Transplant. (2017) 32:1789–805. doi: 10.1093/ndt/gfx002, PMID: 28371878

[29] Che M Wang X Xie B Huang R Liu S Yan Y. Use of both serum Cystatin C and creatinine as diagnostic criteria for cardiac surgery-associated acute kidney injury and its correlation with long-term major adverse events. Kidney Blood Press Res. (2019) 44:415–25. doi: 10.1159/000499647 31189155

[30] Lambermont B D’Orio V. Cystatin C blood level as a risk factor for death after heart surgery. Eur Heart J. (2007) 28:2818. doi: 10.1093/eurheartj/ehm432 17940083

[31] Pilarczyk K Panholzer B Huenges K Salem M Jacob T Cremer J. Prediction of acute kidney injury by Cystatin C and [TIMP-2]*[IGFBP7] after thoracic aortic surgery with moderate hypothermic circulatory arrest. J Clin Med. (2022) 11:1024. doi: 10.3390/jcm11041024, PMID: 35207297 PMC8877349

[32] Johnson EC Muñoz CX Le Bellego L Klein A Casa DJ Maresh CM. Markers of the hydration process during fluid volume modification in women with habitual high or low daily fluid intakes. Eur J Appl Physiol. (2015) 115:1067–74. doi: 10.1007/s00421-014-3088-2 25564016

[33] Engel D Löffel LM Wuethrich PY Hahn RG. Preoperative concentrated urine increases the incidence of plasma creatinine elevation after major surgery. Front Med. (2021) 8:699969. doi: 10.3389/fmed.2021.699969 PMC8327205 34350198

[34] Finge T Bertran S Roger C Candela D Pereira B Scott C. Interest of urinary [TIMP-2] × [IGFBP-7] for predicting the occurrence of acute kidney injury after cardiac surgery: a gray zone approach. Anesth Analg. (2017) 125:762–9. doi: 10.1213/ANE.0000000000002116 28537976

[35] Gocze I Koch M Renner P Zeman F Graf BM Dahlke MH. Urinary biomarkers TIMP-2 and IGFBP7 early predict acute kidney injury after major surgery. PLoS One. (2015) 10:e0120863. doi: 10.1371/journal.pone.0120863, PMID: 25798585 PMC4370650

[36] Honore P Chauwla LS Bihorac A Shaw AD Shi J Kellum JA. Urinary TIMP-2 and IGFBP7 elevate early in critically ill postoperative patients that develop AKI. Crit Care. (2015) 19:P287. doi: 10.1186/cc14367

[37] Schultz-Swarthfigure CT McCall P Docking R Galley HF Shelley B. Can soluble urokinase plasminogen receptor predict outcomes after cardiac surgery? Interact Cardiovasc Thorac Surg. (2021) 32:236–43. doi: 10.1093/icvts/ivaa239, PMID: 33236082 PMC8906672

[38] Tsai HS Chen YC Chu PH. The influence of acute kidney injury on acute cardiovascular disease. Acta Cardiol Sin. (2014) 30:93–7. PMID: 27122774 PMC4805013

[39] Fiorentino M Xu Z Smith A Singbartl K Palevsky PM Chawla LS. Serial measurement of cell-cycle arrest biomarkers [TIMP-2] · [IGFBP7] and risk for progression to death, dialysis, or severe acute kidney injury in patients with septic shock. Am J Respir Crit Care Med. (2020) 202:1262–70. doi: 10.1164/rccm.201906-1197OC, PMID: 32584598 PMC7605192

